# Editorial: Advancements and Challenges in Lung Cancer Screening, Diagnosis, and Management

**DOI:** 10.3390/diagnostics15070835

**Published:** 2025-03-25

**Authors:** Yi-Chi Hung, Yun Lin, Fu-Zong Wu

**Affiliations:** Department of Radiology, Kaohsiung Veterans General Hospital, Kaohsiung 813414, Taiwan; ychuang@vghks.gov.tw (Y.-C.H.); ylin@vghks.gov.tw (Y.L.)

## 1. Diagnostic Challenges and Innovations

### 1.1. High Incidence of False Positives in EGFR S768I Mutation Detection Using the Idylla qPCR System in NSCLC Patients

The study highlights a significant issue in detecting the EGFR S768I mutation using the Idylla qPCR system, with a high false-positive rate. This calls for additional validation using alternative methods, such as next-generation sequencing (NGS), to avoid misclassification and ensure appropriate targeted therapy.

### 1.2. Real-Life Pre-Operative Nodal Staging Accuracy in Non-Small Cell Lung Cancer and Its Relationship with Survival

This multicenter cohort study evaluated the accuracy of preoperative mediastinal nodal staging in NSCLC and its impact on two-year survival. Among the 973 patients, pre- and postoperative staging were concordant in 80%, while 13% were under-staged and 7% were over-staged. Invasive mediastinal staging with EBUS was found to improve staging accuracy. Preoperative understaging was independently associated with higher lung cancer-specific mortality, whereas overstaging had no adverse impact on survival. Accurate nodal staging is crucial for optimal treatment and improved outcomes in patients with potentially curable NSCLC.

### 1.3. Effectiveness of Apparent Diffusion Coefficient (ADC) Values in Predicting Pathologic Subtypes and Grades in NSCLC

Diffusion-weighted MRI results, particularly ADC values, show promise for differentiating NSCLC subtypes and tumor grades. Higher ADC values were linked to lower tumor aggressiveness, which suggests their potential utility in noninvasive tumor characterization.

### 1.4. Rare Driver Mutations in Advanced, Oncogene-Addicted NSCLC: A North Italian Real-World Experience

This registry-based study underscores the importance of comprehensive molecular profiling in advanced NSCLC, as rare driver mutations were identified in a substantial subset of patients. These findings support the expansion of targeted therapy beyond common EGFR, ALK, and ROS1 alterations.

## 2. Advancements in Lung Cancer Screening and Risk Assessment

### 2.1. A Retrospective Analysis: Investigating Factors Linked to High Lung-RADS Scores in a Nonsmoking, Non-Family History Population

This study provides valuable insights into high-risk nodule characteristics among non-smokers without a family history of lung cancer. Identifying novel risk factors could refine the lung-RADS criteria and improve risk stratification in this patient subgroup.

### 2.2. Managing Persistent Subsolid Nodules in Lung Cancer: Education, Decision Making, and Impact of Interval Growth Patterns

Persistent subsolid nodules pose a clinical dilemma, and this study emphasizes the role of patient education and shared decision making. The growth patterns of the nodules influence management strategies and balance early interventions against overdiagnosis.

### 2.3. Predicting the Invasiveness of Pulmonary Adenocarcinomas in Pure Ground-Glass Nodules Using the Nodule Diameter: A Systematic Review, Meta-Analysis, and Validation in an Independent Cohort

The study validated nodule diameter as a predictive marker for invasiveness in pure ground-glass nodules (GGNs), thus, supporting a more individualized follow-up and intervention approach for such lesions.

## 3. Emerging Therapeutic Strategies

### 3.1. Early Effects of Bronchoscopic Cryotherapy in Metastatic NSCLC Patients Receiving Immunotherapy: A Single-Center Prospective Study

The study reports that bronchoscopic cryotherapy provides symptomatic relief and potential synergy with immunotherapy in patients with metastatic NSCLC. Further research is needed to validate the long-term survival benefits.

### 3.2. Improving Outcomes of CT-Guided Malignant Lung Lesion Microwave Ablation by Tract Sealing Using Venous Blood Clot

A novel technique of using autologous venous blood clots for tract sealing post-microwave ablation shows promise in reducing complications such as air leaks and hemorrhage. This approach may improve post-procedural recovery and treatment efficacy.

### 3.3. Usefulness of Saline Sealing in Preventing Pneumothorax After CT-Guided Biopsies of the Lung

The study highlights the effectiveness of saline sealing in reducing pneumothorax incidence post-CT-guided lung biopsies. This simple yet effective technique can enhance procedural safety in routine clinical practice.

## 4. Surgical and Radiation Therapy Considerations

### 4.1. Predictive Value of Clinicopathological Factors to Guide Post-Operative Radiotherapy in Completely Resected pN2-Stage III NSCLC

Identifying patients who benefit most from post-operative radiotherapy (PORT) remains a challenge. This study provides a predictive model that incorporates clinicopathological factors to guide PORT decisions in patients with stage III NSCLC after resection.

### 4.2. Comparison of Outcomes Between Systematic Lymph Node Dissection and Lobe-Specific Lymph Node Dissection for Stage I NSCLC

This comparative analysis of lymph node dissection techniques in early-stage NSCLC suggests that lobe-specific lymph node dissection may offer comparable oncologic outcomes to systematic dissection while reducing surgical morbidity.

## 5. Future Directions in Lung Cancer Research

AI-assisted CAD (Computer-Aided Detection): Advanced artificial intelligence algorithms are utilized to enhance the detection of lung nodules in imaging, reduce inter-reader variability among radiologists, and improve overall diagnostic accuracy. By integrating AI-assisted CAD into routine clinical workflows, early detection rates can be optimized while minimizing false positives and unnecessary follow-up procedures [1,2].

Precision Medicine: expanding comprehensive genomic profiling to tailor personalized treatment plans based on individual tumor characteristics. This approach enables the identification of novel biomarkers, facilitates more effective patient stratification, and improves therapeutic outcomes. By incorporating precision medicine strategies, oncologists can optimize targeted therapies, enhance response rates, and minimize the adverse effects in patients with NSCLC and other malignancies [3].

Strategies to Mitigate Overdiagnosis in the Asian Population: developing and implementing risk-adapted lung cancer screening guidelines tailored to the unique epidemiological characteristics of Asian populations, particularly nonsmokers. This includes refining screening criteria based on genetic predisposition, environmental risk factors, and biomarker-based risk stratification to reduce unnecessary interventions and psychological distress, while ensuring that high-risk individuals receive appropriate early detection and management [4].

## 6. Conclusions

**Figure 1:**
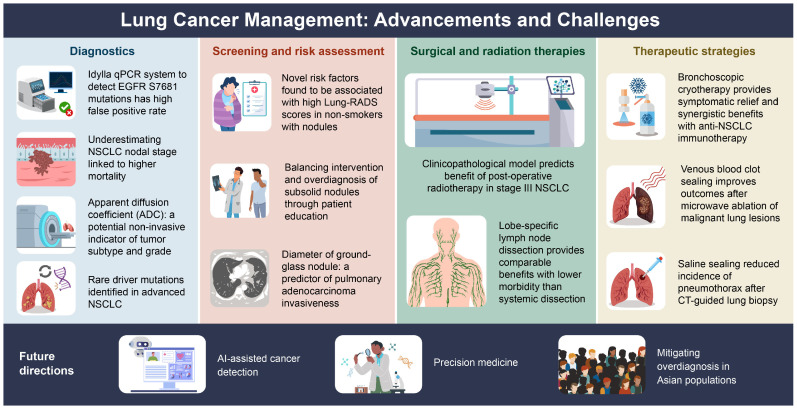
The advancements and challenges in lung cancer management across 12 different domains.

The studies summarized here reflect rapid progress in lung cancer screening, diagnosis, and management shown in Figure 1 and Table 1. However, significant gaps remain, particularly in optimizing screening for nonsmokers, integrating AI into diagnostic workflows, and refining minimally invasive therapies. Future research should focus on precision medicine approaches, AI-driven diagnostics, and balancing early detection while minimizing harm. By addressing these challenges, lung cancer outcomes can be improved, while reducing unnecessary interventions and ultimately enhancing patient care globally. In recent years, Western countries have begun to recognize the importance of lung cancer screening for never-smoking women, as the prevalence of lung cancer in this population continues to rise [5]. Current research on lung cancer screening for never-smokers is primarily conducted in East Asian countries such as Taiwan, China, Japan, and South Korea [6,7]. However, there is a lack of direct evidence from randomized controlled trials (RCTs) on lung cancer screening in Asian never-smokers. Nevertheless, recent evidence-based literature has revealed a significant shift in lung cancer detection among never-smokers undergoing screening [8]. Prior studies have also observed a higher likelihood of overdiagnosis, particularly in never-smokers. Therefore, it is crucial to optimize the effectiveness and quality of lung cancer screening in never-smokers through personalized precision medicine policies and strategies. Future developments are expected to advance clinical lung cancer screening, diagnosis, and treatment toward a more individualized and precision medicine approach [9,10].

The 12 key topics explore the latest advancements and challenges in lung cancer management, including innovations and limitations in diagnostics, emerging risk assessment factors, surgical and radiation therapy strategies, and novel therapeutic approaches like cryotherapy and blood clot sealing. Additionally, future directions emphasize AI-assisted cancer detection, precision medicine, and efforts to reduce overdiagnosis risks, particularly in Asian populations.

## Figures and Tables

**Table 1:** Overview of 12 studies on lung cancer: screening, diagnosis, and management.

Category	Study Title	DOI	Key Findings	Clinical Implications
Diagnostic challenges and innovations	High incidence of false positives in EGFR S7681 mutation detection using the idylla qPCR system in NSCLC patients	10.3390/diagnostics15030321	High false-positive rate in detecting EGFR S768l mutation using idylla qPCR	Validation with NGS is necessary to avoid misclassification and ensure proper targeted therapy
	Real-life pre-operative nodal staging accuracy in non-small cell lung cancer and its relationship with survival	10.3390/diagnostics15040430	Pre-operative nodal staging accuracy was 80%, with 13% under-staged and 7% over-staged. Under-staging linked to higher lung cancer-specific mortality	Emphasizes the importance of invasive mediastinal staging (EBUS) for accurate staging and treatment optimization
	Effectiveness of apparent diffusion coefficient values in predicting pathologic subtypes and grade in NSCLC	10.3390/diagnostics14161795	ADC values correlate with NSCLC subtypes and tumor grades, with higher values linked to lower aggressiveness	Suggests ADC values as a potential non-invasive imaging biomarker for NSCLC characterization
Surgical and radiation therapy considerations	Predictive value of clinicopathological factors to guide post-operative radiotherapy in completely resected pN2-stage III NSCLC	10.3390/diagnostics13193095	Developed a predictive model for guiding PORT decisions in stage III NSCLC	Helps identify patients who would benefit most from PORT.
	Comparison of the outcomes between systematic lymph node dissection and lobe-specific lymph node dissection for stage I NSCLC	10.3390/diagnostics13081399	Lobe-specific lymph node dissection showed comparable oncologic outcomes to systematic dissection with lower morbidity	Supports a more tailored surgical approach to reduce complications
	Predicting the invasiveness of pulmonary adenocarcinomas in pure ground-glass nodules using the nodule diameter: a systematic review, meta-analysis, and validation in an independent cohort	10.3390/diagnostics14020147	Meta-analysis validated nodule diameter as a predictor of invasiveness in GGNs	Supports individualized follow-up and intervention strategies for GGNs
Emerging therapeutic strategies	Early effects of bronchoscopic cryotherapy in metastatic NSCLC patients receiving immunotherapy: a single-center prospective study	10.3390/diagnostics15020201	Demonstrated symptomatic relief and potential synergy with immunotherapy in metastatic NSCLC	Requires further research to validate long-term survival benefits
	Improving outcomes of CT-guided malignant lung lesion microwave ablation by tract sealing using venous blood clot	10.3390/diagnostics14232631	Autologous venous blood clot sealing reduced air leaks and hemorrhage post-microwave ablation	Enhances safety and recovery post-procedure
	Usefulness of saline sealing in preventing pneumothorax after CT-guided biopsies of the lung	10.3390/diagnostics13233546	Saline sealing effectively reduced pneumothorax incidence post-CT-guided biopsy	Simple, cost-effective technique to improve procedural safety
	Rare driver mutations in advanced, oncogene-addicated NSCLC: a north Italian, real-world, registry experience	10.3390/diagnostics14101024	Identified rare driver mutations beyond common EGFR, ALK, and ROS1 mutations in advanced NSCLC	Supports the need for comprehensive molecular profiling to expand targeted therapy options
Advancements in lung cancer screening and risk assessment	A retrospective analysis: investigating factors linked to high lung-RADS scores in a nonsmoking, non-family history population	10.3390/diagnostics14080784	Identified novel risk factors contributing to high lung-RADS scores in non-smokers	Helps refine risk stratification and improve screening strategies fot this subgroup
	Managing persistent subsolid nodules in lung cancer: education, decision making, and impact of interval growth patterns	10.3390/diagnostics13162674	Highlights the importance of shared decision making and monitoring interval growth in persistent subsolid nodules	Balances early intervention and overdiagnosis concerns to optimize patient outcomes
